# Large Polaron Condensation in a Pseudo-Bilayer Quantum Hall Composite

**DOI:** 10.3390/nano14080688

**Published:** 2024-04-16

**Authors:** Bo Dai, Changyue Wang, Junhao Chen, Xin Su, Yuning Shi, Yihan Zeng, Ying Wang, Kai Chen

**Affiliations:** 1 School of Physics, Nanjing University of Science and Technology, Nanjing 210094, China; daibo@njust.edu.cn (B.D.); wcy9264@njust.edu.cn (C.W.); chenjunhao0926@njust.edu.cn (J.C.); suxin@njust.edu.cn (X.S.); zengyihan@njust.edu.cn (Y.Z.); wying813@njust.edu.cn (Y.W.); 2 Department of Physics and Astronomy, University College London, London WC1E 6BT, UK; yuning.shi.22@ucl.ac.uk; 3 MIIT Key Laboratory of Semiconductor Microstructure and Quantum Sensing, Nanjing University of Science and Technology, Nanjing 210094, China

**Keywords:** coupled ferroelectricity and superconductivity, large polaron, pseudo-bilayer quantum Hall system

## Abstract

There is much interest regarding the “coupled ferroelectricity and superconductivity” in the two-dimensional material, bilayer *T_d_*-MoTe_2_; however, the value and the type of electric polarization are unknown. The device structure and the measurement method show that the measured material is the composite of the pseudo-bilayer quantum Hall system, with a thickness of about thirty-six nanometers. The derived dielectric hysteresis loops and the calculated electronic structure reveal that the condensed large polarons are responsible for the reverse ferroelectricity and the coupled superconductivity. The maximum value of polaron-type electric polarization is ~12 nC/μm^2^ or 1.2 × 10^4^ μc/cm^2^.

## 1. Introduction

The ferroelectric field-effect transistor (Fe-FET) is an attractable alternative component for the artificial intelligence (AI) chip, due to the parallel function of data computing and non-volatile storage, the fast read and write speed, and the low power consumption [1,2]. For high integration in advanced semiconductor processing, the nanoscale ferroelectric compound and composite have been intensively studied. Although the ferroelectricity is phenomenologically defined by the shape of the dielectric hysteresis loop, it originates from the electric polarization in inorganic and organic materials with different polar space groups. In these materials, there are partially or fully covalent bondings. The bonding is the overlapping of electron waves, and, intrinsically, it is quantum. Therefore, the electric polarization is the macroscopic quantum effect [3]. The same goes for the superconductivity [4]. The binary coexistence is theoretically possible but experimentally at the cutting edge. Recently, the superconductivity during the switching dynamic process of electric polarization has been suggested in the bilayer *T_d_*-MoTe_2_ [5]. The electric polarization is due to two-dimensional, long-range, and sliding dipoles at the bilayer interface, while the superconducting charge carrier is attributed to paired electrons and holes. The measured device has a cross-section of a few micrometers, and the fabrication is well matched with the current chip manufacturing process. Its function is the same as the Fe-FET’s, but less power is consumed because of the superconducting current and the almost zero electrical resistance. The operating temperature is easily achieved by the dilution refrigerator [6], so the device may be a better choice for neuromorphic computing [7].

**Figure 1:**
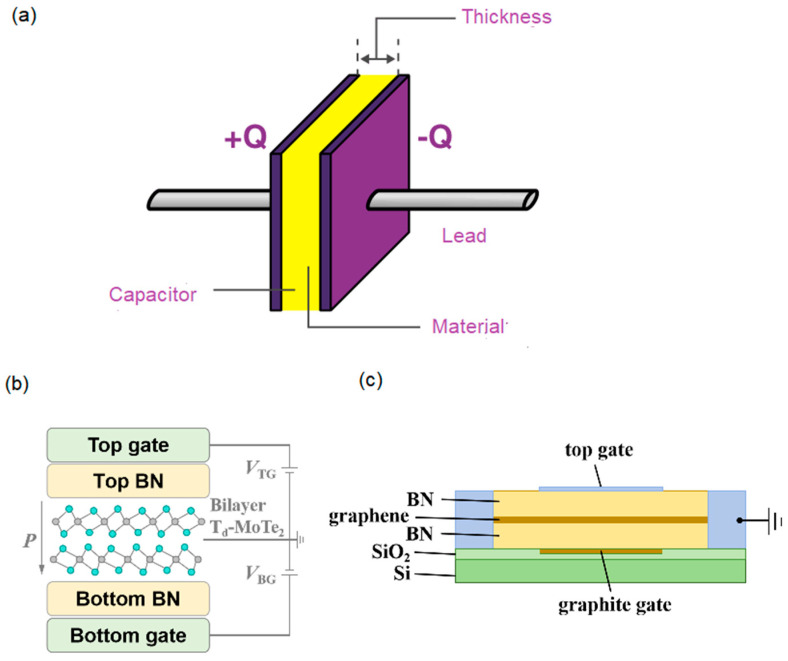
(**a**) The capacitor schematic for the electric polarization measurement. (**b**) The reported schematic in Ref. [5]. (**c**) The cross-section schematic of bilayer quantum Hall system in Ref. [9].

The device structure and the measurement method are the keys to characterize the property. In the measurement method of electric polarization, *P*, the material is usually measured as the internal layer of a parallel-plate capacitor with two metal electrodes, as shown in Figure 1a. When the A.C. voltage of triangular waveform is applied, the switched charged, *Q*, the measured current, *I*, and, *P*, in one period, *T* (1/frequency), and are quantified as the following [3,8]:

(1) P = Q/A = ∫IdT/A

where the *A* is the electrode area. However, the reported device structure [5] consists of the bilayer between two dielectrics of hexagonal boron nitride (hBN), and the top/bottom gate electrodes (Figure 1b). The hBNs at the top and bottom are 29 nm and 7 nm thick, respectively. The structure is the same as that of a bilayer quantum Hall system (BQHS), which is made of a small direct-bandgap semimetal bilayer sandwiched by large bandgap dielectrics [9,10]. The uni-direction current is typically in the electric polarization measurement, while the counter-flowing currents used in the reported measurement method [5] are the same as those used in the BQHS measurement (Figure 1c) [9,10]. However, no quantum Hall phase is observed in the device. After the gate electrodes are excluded, the measured system is not a compound but a nanometer-thickness composite of pseudo-BQHS (p-BQHS). In the composite, there are two-dimensional electron gases (2DEGs) of ~10^13^/cm^2^. The 2DEG of less density in the semiconductor or insulator BQHS shows the ferroelectricity above the liquid helium temperature [11]. It is not surprising that in this semimetal p-BQHS, the denser electron gas has electric polarization above the critical temperature, *T_c_*, of ~2.3 K [4,11].

Exciton-polaron Rydberg states in the hBN-sandwiched monolayer MoSe_2_ and WSe_2_ [12] suggest that the observed superconductivity is a result of electron phase transition, i.e., the large polaron (LP) condensation of 2DEGs. Below *T_c_*, two parallel 2DEGs separated by an atomic bilayer, in part, condense into LPs both within and between layers. The paired LPs comprise polaron-type electrons in one layer bound to holes in the other. During the transporting process, they replace the Cooper pairs as the charge carrier. The thermal energy at *T_c_* [5] suggests that the coupling among the condensed LPs is about 0.20 meV. Below *T_c_*, a collective of LPs behaves as an integral in which the running wave functions and the resultant lattice distortion spreads over the bilayer lattice sites [13]. 

A LP is a type of electron quasiparticle at the self-trapped state due to the induced lattice polarization, while this polarization can follow the charge carrier when it is excited to move through the lattice [13]. Once these charge carriers are paired, the superconductivity appears. The LP is different from the small polaron. From the wave perspective, the electron–lattice interaction and the ground-state wave of a small polaron are confined in a unit cell, while those of the LP exist throughout the entire lattice. From the particle perspective, the mean free path of the excited small polaron is limited in the unit cell, and that of the LP at its ground and excited states is comparable with the lattice scale. On one hand, LPs might be different from band electrons, but they are still the electron quasiparticle. The density function theory (DFT) and the derived simulating calculation are, at present, an effective way to comprehensively reveal the electronic structure, and, thereby, a quantitative mechanism of material properties from the fundamental laws of quantum mechanics. For the strongly correlated electrons, the common approximation in the DFT underestimates the bandgap value, including local density, local spin density, and generalized gradient approximations. In the antiferromagnetic insulator of transitional metal oxide, CaCu_3_Ti_4_O_12_, the strong correlation among the *d* electrons is arbitrarily ignored, and as a result, the band gap is calculated with the characteristic value of a semiconductor ~200 meV. However, the Hubbard on-site correction (DFT+U) improves the value of the direct band gap up to 2.03 eV [14]. There are several improved schemes to account for [15]: the Hubbard on-site correction [16], the self-interaction correction scheme [17,18], and with hybrid [17] or exchange-correlation [19,20] functions. The recent works prove that the electronic structure can be used to profile these strongly correlated electron quasiparticles [21,22]. On the other hand, the polarization type can be distinguished by the shape of the dielectric hysteresis loop, and, simultaneously, the type of charge carrier during the switching dynamic process can be identified by the curve feature of the time-dependent response current. In the study, the DFT+U calculation is performed to investigate the electronic structure. And the time-dependent response current and the dielectric hysteresis loop are derived from the Hall resistance experiments above and below *T_c_*, respectively. All of the results suggest that LPs in the pseudo-ground and -excited states have a key role in the coupled reverse-ferroelectricity and superconductivity.

## 2. Methods

All calculations are performed using the DFT with a planar-augmented basis set as implemented in the Vienna ab initio simulation package (VASP) [23,24]. The localized Wannier functions were performed by using the WANNIER90 package [25,26]. The exchange-correlation term is accounted for within the generalized gradient approximation of the Perdew–Burke–Ernzerhof (PBE) functional [18] revised for solids, PBEsol. The PBEsol exchange-correlation function [20] was used with a cutoff energy of 360 eV. In-plane lattice parameters were relaxed as a = 6.35 Å and b = 3.47 Å [27], and a shifted Monkhorst–Pack grid [28,29] of 8 × 16 × 2000 points was used. Because there is a hybridization of Mo-*d* and Te-*p* orbitals, we introduced the U term on the Mo-*d* orbital to consider the effects of the Coulomb correlations that arise from the more localized nature of this orbital. The quantum oscillation frequencies of MoTe_2_ are measured at about 3.0 eV in the angular resolved photoemission spectroscopy [30], and the coupling energy among the polaron-type electrons is measured at about 88 meV [31]. Therefore, a reasonable value of U is set as 3.1 eV. The DFT+U method has effectively and reliably been proved for the polaron-type electron [21] and MoTe_2_ [30]. The convergence accuracy is less than 1%. We adopted a similar treatment method of the bilayer as Ref. [5].

In the reported sub-quadratic Hall and magnetoresistance measurements [5], the hysteretic loop of the four-probe resistance, *R_xx_*, is measured during the cycling of the displacement field, *D*. The loop actually reveals the charge change in polarization difference, Δ*P*, between different crystalline states [3]. For the quasi-continuous change in *D_n_*, we can derive it from Equation (1) as the following:

Δ*P*_*xx*,*n*_ = *P*_*xx*,*n+1*_ − *P*_*xx*,*n*_ = Δ*Q*_*n*_/*A* = (∆*I*_*xx*,*n*_·∆*t*_*n*_)/*A* = (*U*/∆*R*_*xx*,*n*_·∆*t*_*n*_)/*A* (2)

where *P_xx_*_,*n*_ is the longitudinal polarization in the *n*-th LP excited state, *P_0_* is the initial polarization in the LP ground state, ∆*Q_n_* the transporting charge quantity from the *n*-th to the (*n* + 1)-th LP excited state, *A* is the top gate electrode area, ∆*I_xx_*_,*n*_ the *n*-th response current, ∆*t_n_* the time interval of increasing or decreasing *D_n_*. Based on Equation (2), the dielectric hysteresis loop is achieved from the reported *D_n_*-dependent *R_xx_*_,*n*_ measurement in Ref. [5]. All errors in the calculated results are less than 1%.

## 3. Results and Discussion

**(1) Normal state**

**Figure 2:**
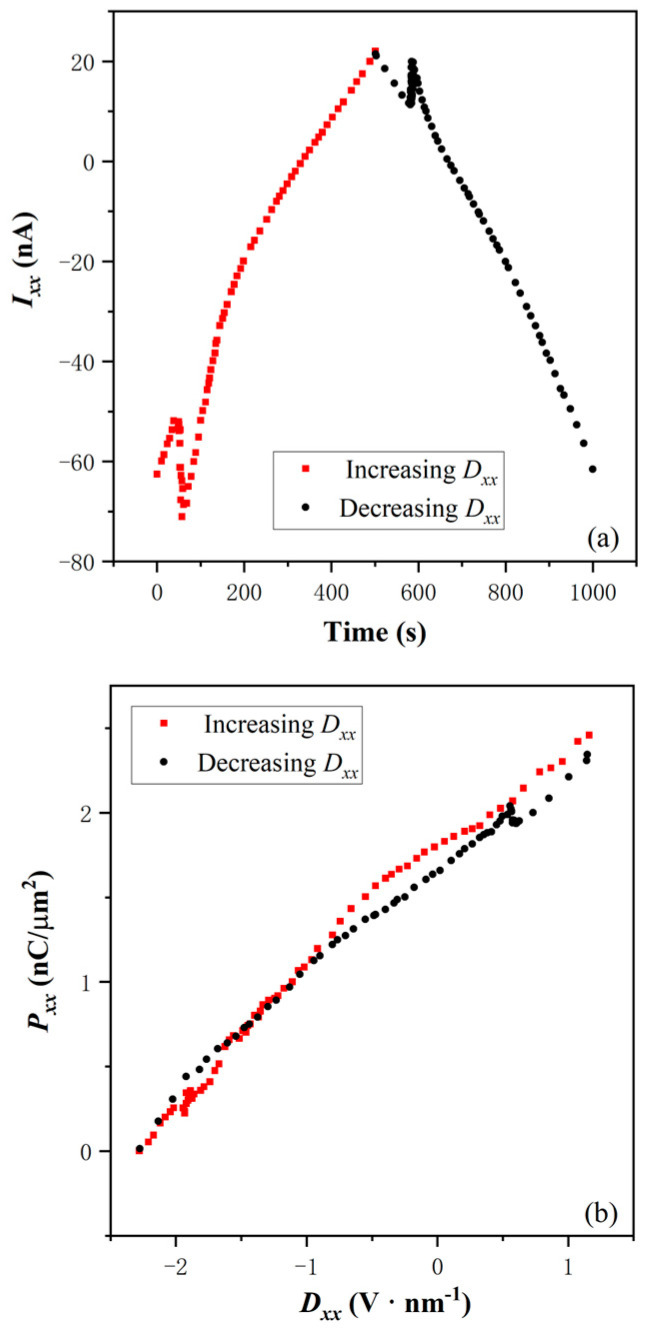
(**a**) Time dependence of *I_xx_* at 4 K. (**b**) Dielectric hysteresis loop of *P_xx_* and *D_xx_* at 4 K.

To identify the electric polarization type, we derive the time-dependent response current (Figure 2a) and the dielectric hysteretic loop (Figure 2b) from the *R_xx_*-*D_xx_* curve above *T_c_*. It is an important step to validate the p-BQHS suggestion. *T_d_*-MoTe_2_ is a type-II Weyl semimetal [32], because the direct bandgap is not zero, and the indirect band gap is zero. When it is between insulating hBN dielectrics, the resultant composite might show good insulation. However, the shape of the dielectric loop does not show the insulating “S”-shape of ferroelectricity. As shown in Figure 2b, the loop is a “banana” shape. Obviously, the curve shape of time-dependent *I_xx_*, is different from the “M”-shape of the ferroelectricity at 4 K. Usually, this is due to the leakage current in the more conductive compound [33]. The 2DEG is more likely responsible for the electric polarization, and self-consistently, as the charge carrier of the leakage current. 

2DEG has been observed in the hBN-based heterostructure. The heterostructure creates a potential well that confines electrons within a narrow region. These electrons are allowed to move freely in the two dimensions within the crystal lattice structure of hBN, in which boron and nitrogen atoms are alternately arranged in a hexagonal lattice plane. In the confined two-dimensional space, quantum mechanical effects become significant. Quantum confinement leads to discrete energy levels for electrons, affecting their behavior, electronic, and electric properties. The hBN heterostructure is different from the semiconductor-dielectric one of the quantum well. In this semiconductor heterostructure, the various dielectric and channel layers are grown epitaxially one by one in a single vacuum environment of 10^−9^ Pa. Electrical contacts to the channel are typically made by diffusing metals into the quantum well.

The *P* is a separation of positive and negative charges within a material. In the context of the 2DEG in the hBN heterostructure, it can be affected by various factors, such as asymmetric heterostructure design, quantum capacitance, gate voltage tuning, quantum Hall effect, and so on. In the reported hBN composite [5], top and bottom hBN dielectrics of different thicknesses are used to achieve an asymmetric design to set the maximum value of electric polarization. Because the quantum capacitance is related to the change in charge density with respect to the potential, it might be influenced by the electronic structure of the 2DEGs and the intermediate bilayer *T_d_*-MoTe_2_. Applying a gate voltage to the composite may modify the charge density to regulate the carrier concentration during the switching dynamic process. In the presence of a magnetic field, the 2DEG in other hBN heterostructures can exhibit the quantum Hall effect. This effect results in the quantized Hall conductance and can lead to dielectric hysteresis loops with a discontinuous characteristic. However, the reported composite [5] does not show this effect, which is perhaps due to its unusually denser electron gases.

**(2) Superconducting state**

**Figure 3:**
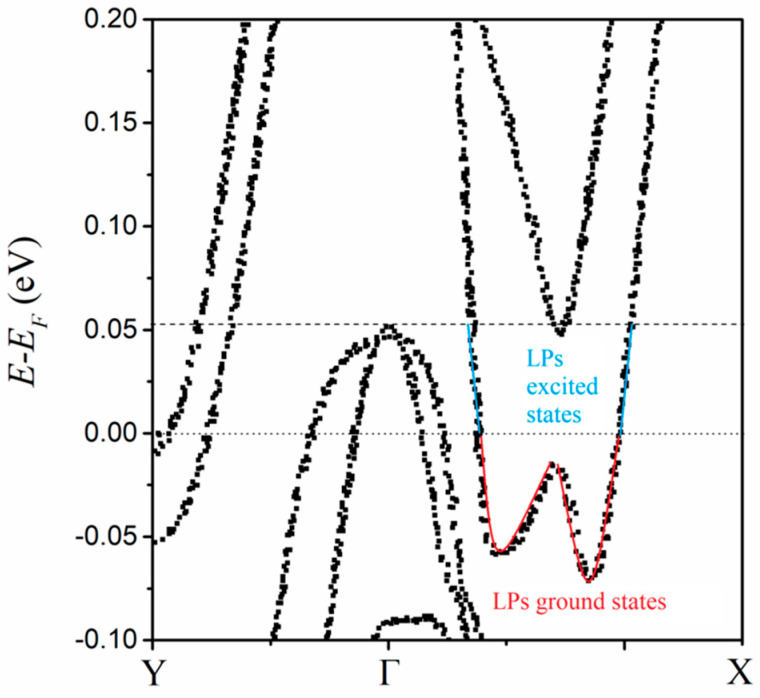
Electronic structure of LPs condensed in bilayer *T_d_*-MoTe_2_ lattice.

The calculated electronic structure is similar to the reported one [5]. The difference is that the states are not continuous but discrete (Figure 3), which is consistent with the limited quantity of condensed large polarons and the hopping way during the transport process [13]. The discrete state may be responsible for the quantum capacitance. This quantum characteristic of electronic structure is consistent with the nature of 2DEG. LPs condense into the lattices of the *T_d_*-MoTe_2_ bilayer below *T_c_*, and as a result, the bilayer can be regarded as the doping by these specific electron quasiparticles. As a strongly correlated system, these electron quasiparticles induce lattice distortion for the electric polarization. At the same time, they may modify the crystal field, result in the disappearance of the direct bandgap, and trigger the appearance of the indirect band gap.

The bilayer is between the top and bottom dielectrics. These dielectrics can function as the charge reservoirs for the non-saturating magnetoresistance [5]. The two dielectrics of different nano-thicknesses produce the electron gases of different concentrations above the top layer and below the bottom layer, respectively. When the temperature is decreased below *T_c_*, there is a density difference in LPs between the top layer and the bottom one. The difference induces anisotropic and inhomogeneous distortions in the bilayer lattice. The distortion relaxes the lattice to different degrees, and it is artificial in the calculation to relax the internal ionic coordinates. The relaxed lattice prompts multi-orbital hybridization, which results in the asymmetricity of the electronic structure. Because the dielectrics continuously supply electrons, LPs’ condensation of 2D electron gases can provide enough electrons to fill the pocket of the conduction band bottom below the Femi surface (FS). The FS pocket shows an asymmetric double-well shape (the red line in Figure 3). Notably, the double potential well is a typical model for the switching dynamics of electric polarization. For the switching subprocess of electric polarization, the states in the FS pocket are considered the pseudo-ground states or LP ground states. The hole density can be decreased by the doping and the resultant vanishment of *T_c_* is accompanied by the disappearance of the FS pocket [5]. Therefore, the paired holes are considered to occupy the excitation-induced vacant states in the cone of the valence band top above the FS. Then, the pseudo-excited states or LP excited states are between the cone top and the FS surface (the blue line of Figure 3).

The interstate hopping among these pseudo-ground states can lead to the switching dynamic of *P* in a quasi-continuous way. During the subprocess, LPs in the pseudo-ground states are driven by the applied electric field, and via the inherent coupling, successively occupy the next pseudo-ground states along the line of the FS pocket (the red line in Figure 3). At the same time, the same quantity of LPs is pushed out of the FS pocket to occupy the pseudo-excited states (the blue line in Figure 3), and behave as the charge carrier of Δ*P* between different crystalline states. As a result, some LP ground states become vacant. For the lowest energy favor of the system, the electrons in the cone of valence band top over the FS, hop to occupy the vacant LP ground states. Simultaneously, the result holes are created for the pairing in the superconducting current. Obviously, the superconductivity disappears when no hole carriers are present, and they can reemerge when they are reintroduced [5].

**Figure 4:**
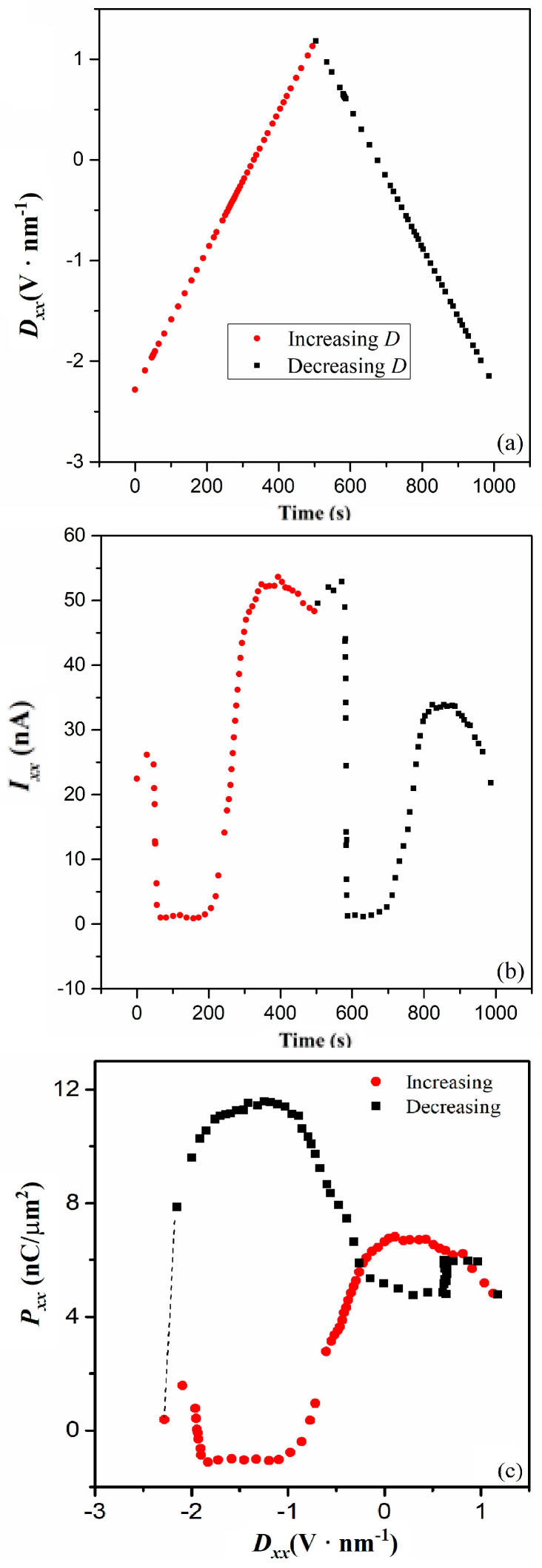
(**a**) Time dependence of *D_xx_* at 1.7 K. (**b**) Time dependence of *I_xx_* at 1.7 K. (**c**) Dielectric hysteresis loop of *P_xx_* and *D_xx_* at 1.7 K.

The response current, *I_xx_*, during the switching dynamic subprocess, and the resultant dielectric hysteresis loop, is examined to confirm that the LPs are simultaneously involved in both the superconducting current and the switching dynamic of *P*. The time dependences of *D_xx_* and *I_xx_* are derived from the *D_xx_* dependence of *R_xx_* in Ref. [5]. As shown in Figure 4a, the time dependence of *D_xx_* shows the curve of a triangle wave, which is similar to that of the electric polarization measurement method. Notably, the current in the reported measurements is up to 100 nA [5]; however, the exciton effect and the pairing effect do not change the response current range of the switched electric polarization, *I_xx_*. (Figure 4b). When the *D_xx_* is increased, and, then decreased, the time dependence of the resultant *I_xx_* shows an unusual curve, which is parallel time axis asymmetric and doorframe shaped. The curve characteristic reveals the electric polarization is not ionic but a polaron type [8]. Consistently, the hysteresis loop of *P_xx_* and *D_xx_* shows the abnormal reverse S-shape (Figure 4c), which is a sign of polaron-type polarization [8]. The maximum value of *P_xx_* is ~12 nC/μm^2^ or 1.2 × 10^4^ μc/cm^2^, and notably, far larger than that of the well-known ionic polarization, which often shows the value of ~1.5 × 10^2^ μc/cm^2^. A very large value of electric polarization ~ 3 × 10^4^ μc/cm^2^ is reported in a novel organic−inorganic hybrid [34]. The switching dynamic of *P* is attributed to proton migration. To calculate the migration energy barrier of proton migration, DFT calculations have also been performed with the VASP, employing the projector-augmented wave pseudopotentials, the PBE exchange−correlation functional, and the climbing image-nudged elastic band method [34]. The calculation results indicate that the energy barrier for the proton migration follows the sequence//b-axis <//c-axis <//a-axis. This sequence is consistent with experimental observations. In addition, the *D_xx_* independence of the maximum *P_xx_* is the same as that of quasipolaron surface polarization [6]. Thus, the charged particles that are smaller than ions can contribute to larger electric polarization.

We conclude that the charge carriers for the switching dynamic subprocess are “mobile” LPs in pseudo-excited states, and the electric polarization originates from “localized” LPs in pseudo-ground states. The asymmetric ground-state double well results in the asymmetry of a hysteresis loop. In the superconducting subprocess, *P_xx_* is decreased to 1 nC/μm^2^ because of the fewer “localized” LPs. Ferroelectric LPs have been proposed in lead halide perovskites [35]. The local electric field is intense enough to induce the collective behavior of electric polarization, and it triggers local phase transitions to form and order the polar nanodomains surrounding the charge. The result is a ferroelectric LP. When charge carriers in ferroelectric LPs begin to hop between their ground states, dynamic symmetry breaking and local alignment of electric polarization may be induced. This dynamical disorder alters the potential energy surface for atomic displacements in a unit cell, which shows multiple minima rather than a single minimum close to the equilibrium position. As a result, under sufficiently intense electric fields, the *P* can flip between different minima, changing in sign, and increasing by one or more orders of magnitude in absolute value. The *D_xx_*-dependent response of *P_xx_* can also show a dielectric hysteresis loop if the potential barrier between the two minima is above the thermal energy *kT*. In the calculated electronic structure, the potential barrier in the double well at about 0.025 eV is far larger than the thermal energy of 0.20 × 10^−3^ eV at *T_c_*. Therefore, a similar switching dynamic process can happen below *T_c_*. Just like ferroelectric LPs, the LPs may be characterized by analyzing the dielectric response in the terahertz region to study how such phonon behavior affects the formation and properties of LPs. They can be also characterized by the longitudinal optical phonon-absorption spectrum because the LPs are charge-coupling with these phonons. The low-frequency Raman spectra can be used for revealing the existence of LPs because they result in the soft-mode behavior of the quasi-elastic central peak near-zero frequency. 

To analyze the electric polarization, we combine the DFT+U with the polaron theory, because the polaron is the electron quasiparticle. The DFT has been combined with other theories to deal with the complex physic properties, including the ab initio molecular dynamics theory, the self-consistent phonon theory, the dynamical mean-field theory within the linear combination of numerical atomic orbital basis set framework, and so on. The DFT is, at present, a powerful and widely used quantum mechanical modeling method. The key quantity is not the wave function but the electron density. The wave function is used in traditional approaches to quantum mechanics, while the electron density is a spatial distribution of electron charge in a system. As the base of DFT, the Hohenberg–Kohn theorems state that the ground-state electron density uniquely determines the ground-state wave function and the surrounding potential, when the exchange-correlation potential is excluded. The fundamental equations are the Kohn–Sham, in which a set of fictitious non-interacting electrons with an effective potential is introduced. The Kohn–Sham orbitals represent an auxiliary system, whose electron density approximates the true electron density of the interacting system. The DFT offers some important advantages. Firstly, it is computationally more efficient compared to traditional quantum mechanical methods, such as the Hartree–Fock theory. This efficiency allows us to study the complex system within reasonable computational resources. Secondly, it provides a practical and accurate way to describe the electronic structure of many-body systems, and allows for the study of materials with a large number of interacting electrons. Finally, it provides good quantum mechanical accuracy for the system. However, it is important to note that DFT has limitations. Its accuracy depends on the choice of exchange-correlation functional and the nature of the system under investigation. The exchange-correlation functional is a crucial component, representing the effects of electron–electron exchange and correlation. It combines the exchange and correlation energies into a functional form. Approximations for this functional are often employed, as obtaining an exact form is challenging. 

Our investigation shows some areas in which the DFT can improve. Firstly, we assume that the electronic structure is stable in the temperature range from 0 K to 2.3 K because the thermal energy is smaller than the 1% of U energy. The thermal parameter may be added to the DFT. Secondly, the U value seems artificial, although it is chosen based on the experiments. We assume that all the unit cells are the same, regardless of their quantity. However, the relaxation degree depends on the quantity of unit cells in the real crystalline structure. The same goes for the exchange and correlation energies, the kinetic energy, electron–electron interaction, and external potentials. The determined crystalline structure results in the corresponding crystalline state with a specific U, and this U may give us the exact value. We hope that the DFT will be developed to self-consistently reveal the U value by optimizing the calculation method of the crystalline structure. Moreover, the total energy is minimized with respect to variations in the electron density to find the ground-state properties of the system, and other physics parameters may be ascertained in a similar way with the assistance of AI. Thirdly, we have assumed that the electrical excitation does not change the electronic structure but rather excites electrons or electron quasiparticles in the given states. We hope the threshold value of the stable electronic structure will be revealed when strain, thermal, electrical, magnetic, and photon fields are applied. Finally, we can do nothing to find out the time response of the dielectric function, and, therefore, we hope that the Kohn–Sham equations will be modified by using the time-dependent Hamiltonian equation. The time-dependent equation will help us describe the response to A.C. electric fields. It can practically interpret experimental results by providing a theoretical framework, and identify the underlying electronic mechanisms that govern the material properties. 

## 4. Conclusions

The temperature-dependent Hall resistance suggests that some 2D electron gases condense into large polarons below a critical temperature. We examine the band structure of LPs by using the density functional theory with the Hubbard on-site Coulombic correction. The electronic structure shows that there is a double-well-shaped pocket of the lowest conduction band below the Fermi surface (FS). The dielectric hysteresis loop of reverse S-shape is derived from the Hall resistance measurement, signifying reverse ferroelectricity and a large electric polarization ~12 nC/μm^2^. The discontinuous state in the FS pocket and the loop, in a self-consistent way, reveal that LPs are electrically driven to hop among pseudo-ground states, accompanied by the switching dynamic of induced lattice polarization. Others are pushed to hop in pseudo-excited states and pair with the holes in the cone of the highest valence band over the FS, both of which behave as superconducting charge carriers. The inherent coupling among these LPs correlates with the electric polarization and the superconductivity at the macroscopic scale. 

## Data Availability

Data are contained within the article.
